# Safety of Proton Pump Inhibitor in Paediatrics: A Study Based on EudraVigilance Data

**DOI:** 10.1111/fcp.70036

**Published:** 2025-07-07

**Authors:** Greta Santi Laurini, Victoria Nikitina, Nicola Montanaro, Domenico Motola

**Affiliations:** ^1^ Unit of Pharmacology, Department of Medical and Surgical Sciences Alma Mater Studiorum University of Bologna Bologna Italy; ^2^ Pharmacology at the Alma Mater Studiorum University di Bologna Bologna Italy

**Keywords:** adverse drug reactions, children, pharmacovigilance, proton pump inhibitors

## Abstract

**Background and Objectives:**

Despite limited paediatric approvals, the use of proton pump inhibitors (PPIs) among children has increased in recent years, and concerns have arisen about their safety, including the risk of allergies. To provide additional evidence on the paediatric safety of PPIs, we performed a study on suspected adverse drug reactions (ADRs) in clinical practice.

**Methods:**

We retrieved from EudraVigilance reports of suspected ADRs for PPIs in the age class 0–11 years in the period 2003–2022. Most reported ADRs and allergic conditions were evaluated by descriptive analysis. A case‐non‐case analysis was performed using reporting odds ratio (ROR) with a 95% confidence interval (CI). Chi‐square or Fisher's exact tests were used to detect differences in reporting rates.

**Results:**

A total of 1389 reports and 4157 suspected ADRs were analysed. Most reports involved omeprazole (46.9%) and esomeprazole (27.3%), and 75.8% concerned serious outcomes. Gastrointestinal disorders were among the most common suspected ADRs, with vomiting being the most frequently reported (2.3%). Among allergic conditions, there were six cases of toxic epidermal necrolysis, five of Stevens–Johnson syndrome and four of drug reaction with eosinophilia and systemic symptoms. Statistically significant reactions for omeprazole were choking (ROR 5.54; 95% CI, 1.04–29.56) and pneumonia (3.61; 1.41–9.20), while for esomeprazole gastrointestinal disorder (7.57; 1.18–48.60) and constipation (4.74; 2.25–9.98).

**Conclusions:**

Most common suspected ADRs reported with paediatric PPI use in Europe were gastrointestinal disorders, consistent with the safety profile in the product license. However, a high proportion of serious ADRs and few cases of severe cutaneous adverse reactions were reported.

**Abbreviations**
ADR: adverse drug reaction
CI: confidence interval
CYP: cytochrome P450
EEA: European Economic Area
EMA: European Medicines Agency
EU: European Union
EV: EudraVigilance
GERD: gastroesophageal reflux disease
HLGT: High‐Level Group Term
HLT: High‐Level Term
ICH: International Conference on Harmonisation
Ig: Immunoglobulin
LLT: Lowest Level Terms
MedDRA: Medical Dictionary for Regulatory Activities
PPI: proton pump inhibitor
PT: Preferred Term
ROR: Reporting odds ratio
SCAR: severe cutaneous adverse reactions
SOC: System Organ Classes
SPC: Summary of the Product Characteristics

## 1 Introduction

Proton pump inhibitors (PPIs) are among the most frequently used medicines off‐label in paediatrics throughout the European Union (EU) [1]. Esomeprazole and omeprazole are the only PPIs approved in Europe for patients over 1 year of age, and dexlansoprazole and pantoprazole are authorised for those above the age of 12.

The use of PPIs in the paediatric population has progressively risen in Europe over the past three decades [2, 3, 4]. In a nationwide drug utilisation study among Danish children and adolescents, the total annual use of PPIs increased eight times from 2000 to 2015 [3], confirming the trend of recent years. Despite the lack of demonstrated efficacy and the presence of guidelines cautioning about the use of PPIs in younger children, a significant increase in PPI prescriptions has also been reported among newborns and infants worldwide [5, 6, 7, 8, 9]. While the beneficial role of PPIs in older children is somewhat controversial, there is no evidence of efficacy in the treatment of non‐specific symptoms attributed to gastroesophageal reflux disease (GERD) in children under 1 year of age [10, 11]. Even though symptoms such as irritability, crying, cough, vomiting or regurgitation are commonly described in patients with GERD, a weak association between acid reflux and these manifestations was found in infants [12, 13, 14], discouraging the use of PPIs in the absence of a documented acid‐related condition.

Along with the increasing use of PPIs in the paediatric population, concerns about the safety of PPIs have been raised in recent years, including an increased risk of infections, bone fractures and allergies [15]. Acid suppressive medications may enhance the development of allergic disorders through the alteration of the human microbiome, intimately linked to a healthy immune system, and through the interference with protein digestion in the stomach, which may lead to an increased sensitisation to ingested antigens [16]. Among allergic diseases, recent cohort studies on the safety of PPIs in paediatric patients have investigated the potential association between PPI exposure and the occurrence of asthma, all highlighting a significantly increased risk of developing asthma among children treated with PPIs [16, 17, 18].

Given the growing paediatric use of PPIs together with the limited supporting data available, a deeper understanding of PPI adverse effects in children is critical for the evaluation of the benefit/risk profile. The most appropriate tool for the continuous evaluation of the safety profile of drugs that have been in clinical use for many years is represented by pharmacovigilance and the spontaneous reporting of suspected adverse drug reactions (ADRs) by healthcare personnel. Spontaneous reporting systems, such as that of EudraVigilance (EV), allow the early detection of potential safety signals and ongoing assessment of potential risks related to reported ADRs. Post‐marketing data reflect real‐world patient populations, since they include those excluded from clinical trials, such as vulnerable groups, patients with comorbidities and/or with multiple medications.

With the aim of providing further evidence on the safety profile of PPIs, a surveillance study was performed to assess all suspected ADRs reported with PPIs in paediatric patients in EudraVigilance (EV).

## 2 Material and Methods

Data on paediatric PPI use were retrieved from the European database of suspected ADR reports EudraVigilance, using the publicly accessible portal adrreports.eu [19]. Launched in December 2001, EudraVigilance is the system designed to collect, manage and analyse suspected adverse reactions to medicines that have been authorised or are being studied in clinical trials in the European Economic Area (EEA). With the aim of supporting the safe and effective use of medicines, EudraVigilance enables the early detection of potential safety issues, providing an invaluable tool for public health protection [20]. The present safety surveillance study was performed by retrieving all reports of suspected ADRs related to PPIs (dexlansoprazole, esomeprazole, lansoprazole, omeprazole, pantoprazole and rabeprazole) as suspect drugs in the age class 0–11 years. The database was browsed by name of individual active substance and over a period of 20 years, from 1st of January 2003 to 31st December 2022.

### 2.1 Descriptive Analysis

Reports from EudraVigilance were identified by a unique EU local number and included information such as EV gateway receipt date, primary source qualification, patient demographic characteristics, a list of suspect/interacting and concomitant/not administered drugs and a description of reported suspected reactions, including duration, outcome and seriousness. Paediatric patients were categorised, according to the International Conference on Harmonisation (ICH) Guideline [21], into the following age groups: preterm newborn infants, term and post‐term newborn infants (0 to 27 days), infants and toddlers (28 days to 23 months) and children (2 to 11 years). According to the European Medicines Agency (EMA) guideline on good pharmacovigilance practices [22], serious adverse reactions corresponded to any medical occurrence that resulted in death, was life‐threatening, required inpatient hospitalisation or prolongation of existing hospitalisation, resulted in persistent or significant disability or incapacity, was a congenital anomaly/birth defect or resulted in another medically important condition. Suspected reactions were entered into EudraVigilance using the Medical Dictionary for Regulatory Activities (MedDRA) [23], a rich and highly specific standardised medical terminology to facilitate the sharing of regulatory information internationally for medical products used by humans. MedDRA terminology consists of a five‐tiered multi‐axial hierarchy, which provides increasing specificity as one descends it [24]. At the top level are the System Organ Classes (SOCs), which are linked to the more specific lower groupings High Level Group Terms (HLGTs) and High Level Terms (HLTs). The single medical concept level of MedDRA is the Preferred Term (PT) [23], a distinct descriptor for a symptom, sign, disease diagnosis, therapeutic indication, investigation, surgical or medical procedure and medical social or family history characteristic that is related to one or more HLTs. Finally, the Lowest Level Terms (LLTs) include synonyms, lexical variants or alternative spellings linked to each PT [25]. Each report in EudraVigilance may include one or more PTs, resulting in the number of suspected ADRs exceeding the number of reports. Furthermore, several drugs, including different PPIs, may be reported as suspect drugs, resulting in multiple drug‐reaction pairs for a single report. For individual PPIs, most reported drug‐reaction pairs were evaluated by performing a descriptive analysis and by assessing the notoriety according to the corresponding Summary of the Product Characteristics (SPCs) [26, 27, 28, 29, 30, 31]. In light of the latest safety concerns, all reactions under the MedDRA HLGT of allergic conditions were retrieved and analysed. Given the multi‐axial structure of MedDRA terminology, all HLGT locations of PTs were considered for this purpose.

### 2.2 Statistical Analysis

For suspected ADRs reported with a frequency ≥ 2 (as per 2016 EMA guideline Screening for adverse reactions in EudraVigilance, https://www.ema.europa.eu/en/documents/other/screening‐adverse‐reactions‐eudravigilance_en.pdf), a case‐non‐case analysis was performed using the reporting odds ratio (ROR) with 95% confidence interval (CI) as a statistical parameter. ROR is a disproportionality analysis designed to compare the frequency of a drug‐related event with the frequency of the same event reported with all other products in the database. Since the aim was to assess the safety of PPIs among children, the analysis was carried out by comparing the occurrence of a drug‐reaction pair related to a single PPI with drug‐reaction pairs related to all other PPIs in paediatric patients. Chi‐square or Fisher's exact tests, as appropriate, were used to assess whether there was a statistically significant difference in the number of suspected PPI‐related reactions. Data management and calculation were performed by using Microsoft Excel, a software developed by Microsoft Corporation.

## 3 Results

Between 2003 and 2022, a total of 1458 reports of suspected ADRs related to the use of PPIs among patients aged 0–11 years were submitted to EudraVigilance. Reports with PPIs listed as interacting drugs or for which the suspect/interacting drug list was not available were excluded, resulting in a total of 1389 reports and 4157 reactions analysed in this study. For both descriptive and statistical analysis, we excluded PTs that were not evaluable as suspected ADRs, such as product use issue, product prescribing error or product formulation issue.

### 3.1 Descriptive Analysis

**FIGURE 1:**
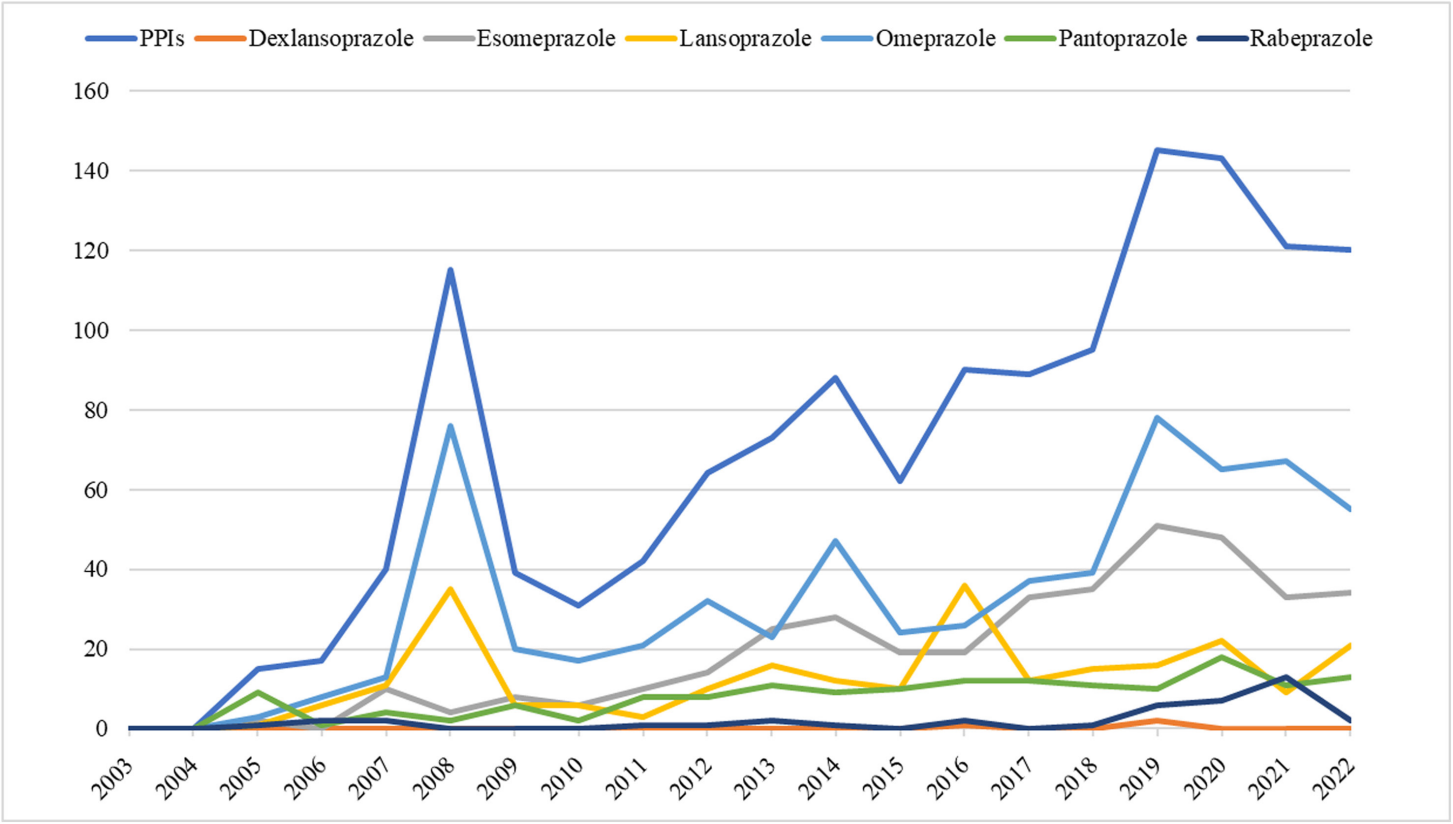
Number of reports involving the paediatric use of proton pump inhibitors (PPIs) submitted to EudraVigilance during the study period.

The number of reports of suspected ADRs involving the paediatric use of PPIs progressively increased over the study period, with peaks recorded in 2008 and 2019 (Figure 1). With regard to primary source qualification, almost 80% of reports were submitted by healthcare professionals. Among all 1389 reports included in the study, dexlansoprazole was reported as a suspect drug in three (0.2%) reports, esomeprazole in 379 (27.3%), lansoprazole in 247 (17.8%), omeprazole in 651 (46.9%), pantoprazole in 157 (11.3%) and rabeprazole in 41 (3.0%).

#### 3.1.1 Dexlansoprazole

Reports of suspected ADRs related to dexlansoprazole concerned three females, one for each age category analysed. A total of six reactions were reported following the paediatric use of dexlansoprazole, all described as medically important conditions. Renal disorders accounted for half of the reactions, including two cases of chronic kidney disease and one case of renal injury.

#### 3.1.2 Esomeprazole

**FIGURE 2:**
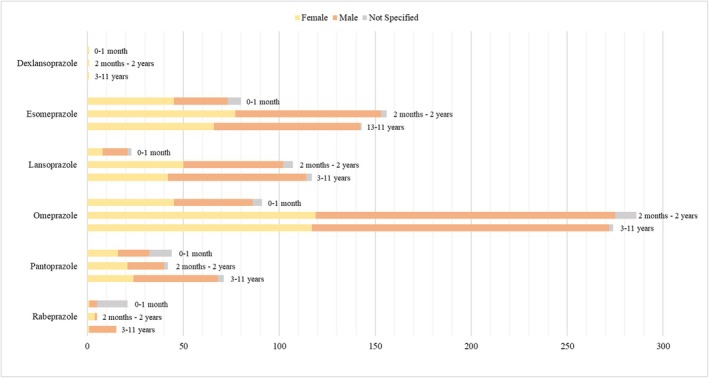
Demographic characteristics of patients expressing suspected ADRs for individual PPIs.

Reports on esomeprazole involved a total of 188 (49.6%) females and 180 (47.5%) males. Overall, 156 (41.2%) patients were between 2 months and 2 years, 143 (37.7%) between 3 and 11 years and 80 (21.1%) less than 1 month (Figure 2 and Table S1 in the Supplementary Appendix). There was a total of 1117 suspected reactions associated with esomeprazole, 69.0% of which were described as serious. The top four most common reported reactions were vomiting (26; 2.3%), drug ineffective (16; 1.4%), constipation (14; 1.3%) and diarrhoea (14; 1.3%) (Table 1 and Table S2 in the Supplementary Appendix). Among patients who received esomeprazole, a total of 14 (3.7%) deaths occurred, five of which involved esomeprazole as the only suspect drug (Table S3 in the Supplementary Appendix).

**TABLE 1:** Suspected ADRs reported in EudraVigilance with frequency ≥ 10 for individual PPIs.

Esomeprazole		Lansoprazole		Omeprazole		Pantoprazole		Rabeprazole	
Events	*n* (%)^a^	Events	*n* (%)	Events	*n* (%)	Events	*n* (%)	Events	*n* (%)
Vomiting	26 (2.3)	Vomiting	20 (2.3)	Vomiting	44 (2.3)	Premature baby	16 (4.0)	Premature baby	18 (8.4)
Drug ineffective	16 (1.4)	Rash	13 (1.5)	Hypertrichosis	35 (1.9)			Trismus	18 (8.4)
Constipation	14 (1.3)	Seizure	13 (1.5)	Diarrhoea	34 (1.8)			Drug withdrawal syndrome neonatal	17 (7.9)
Diarrhoea	14 (1.3)	Therapy non‐responder	12 (1.4)	Drug ineffective	22 (1.2)			Crying	13 (6.0)
Agitation	13 (1.2)	Drug ineffective	11 (1.3)	Abdominal pain	18 (1.0)			Sleep disorder	13 (6.0)
Gastrooesophageal reflux disease	12 (1.1)	Malaise	11 (1.3)	Pyrexia	18 (1.0)			Tremor	13 (6.0)
Abdominal pain	11 (1.0)	Lower respiratory tract infection	10 (1.2)	Rash	17 (0.9)			Muscle tone disorder	12 (5.6)
Insomnia	10 (0.9)	Pyrexia	10 (1.2)	Nausea	15 (0.8)				
Pyrexia	10 (0.9)	Urticaria	10 (1.2)	Dyspnoea	13 (0.7)				
Regurgitation	10 (0.9)			Headache	13 (0.7)				
				Pneumonia	13 (0.7)				
				Sputum discoloured	13 (0.7)				
				Abdominal pain upper	12 (0.6)				
				Gastrooesophageal reflux disease	12 (0.6)				
				Premature baby	12 (0.6)				
				Pancreatitis	11 (0.6)				
				Seizure	11 (0.6)				
				Infantile spitting up	10 (0.5)				
				Regurgitation	10 (0.5)				
				Somnolence	10 (0.5)				
				Urticaria	10 (0.5)				

^a^ Of the total number of suspected ADRs for individual PPIs.

#### 3.1.3 Lansoprazole

For lansoprazole, the number of reports concerning male patients accounted for more than a half, totalling 137 (55.5%) reports. The majority of patients (224; 90.7%) were from 2 months to 11 years old, while 23 (9.3%) were younger than a month (Figure 2 and Table S1). Overall, 864 suspected adverse reactions associated with lansoprazole occurred, and as many as 88.3% were rated as serious. As shown in Tables 1 and S2, the most frequently reported reactions included vomiting (20; 2.3%), rash (13; 1.5%) and seizure (13; 1.5%). Out of six (2.4%) reports of death, four were related to lansoprazole as the only suspect drug (Table S3).

#### 3.1.4 Omeprazole

Reports of suspected ADRs to omeprazole concerned a prevalence of males, with a total of 352 (54.1%) patients. Most paediatric patients (560; 86.0%) were distributed in the age range of 2 months to 11 years, while 91 (14.0%) were under 1 month old (Figure 2 and Table S1). A total of 1886 reactions occurred after administration of omeprazole, with vomiting (44; 2.3%), hypertrichosis (35; 1.9%) and diarrhoea (34; 1.8%) being the most reported (Tables 1 and S2). Overall, 78.4% of all reported reactions resulted in serious outcomes, and a total of 24 (3.7%) patients died. For seven of them, omeprazole was reported as the only suspect drug (Table S3).

#### 3.1.5 Pantoprazole

Of the reports related to pantoprazole, 79 (50.3%) involved males, 61 (38.9%) females and 17 (10.8%) lacked information on sex. A total of 71 (45.2%) patients belonged to the 3–11‐year class, while the remainder was uniformly distributed between the 0–1 month (44; 28.0%) and 2 months–2 years (42; 26.8%) categories (Figure 2 and Table S1). Overall, 400 suspected adverse reactions were reported for pantoprazole, 79.8% of which were described as serious.

#### 3.1.6 Rabeprazole

For rabeprazole, the proportion of reports involving males was higher than for females, with a total of 19 (46.3%) versus 6 (14.6%) reports of suspected ADRs. However, for 16 (39.0%) reports, information on sex was missing. With regard to age, more than half of the patients were less than 1 month old, making a total of 21 newborn infants (51.2%) (Figure 2 and Table S1). Of all 215 reactions for rabeprazole, premature baby (18; 8.4%) and trismus (18; 8.4%) were the most commonly reported (Tables 1 and S2), and adverse reactions with serious outcomes accounted for 91.6%.

#### 3.1.7 Allergic Conditions

Among all 4157 suspected adverse reactions related to paediatric PPI use, a total of 113 (2.7%) were linked to allergic conditions according to the MedDRA classification. As shown in Table 2, the most reported reaction was urticaria (25; 0.6%), followed by hypersensitivity (11; 0.3%) and drug hypersensitivity (7; 0.2%). Potentially life‐threatening allergic conditions included six cases of toxic epidermal necrolysis, five of Stevens–Johnson syndrome and four of drug reaction with eosinophilia and systemic symptoms.

**TABLE 2:** Suspected ADRs under the MedDRA HLGT of allergic conditions reported in EudraVigilance with frequency ≥ 2 for PPI.

Reactions	*n*	%^a^
Urticaria	25	0.60
Hypersensitivity	11	0.26
Drug hypersensitivity	7	0.17
Angioedema	6	0.14
Toxic epidermal necrolysis	6	0.14
Asthma	6	0.14
Stevens‐Johnson syndrome	5	0.12
Swelling face	5	0.12
Drug reaction with eosinophilia and systemic symptoms	4	0.10
Face oedema	4	0.10
Laryngeal oedema	4	0.10
Lip swelling	4	0.10
Food allergy	3	0.07
Bronchospasm	3	0.07
Anaphylactic shock	3	0.07
Anaphylactic reaction	2	0.05
Dermatitis allergic	2	0.05
Drug eruption	2	0.05

^a^ Of the total number of suspected ADRs analyzed.

### 3.2 Statistical Analysis

For the disproportionality analysis, all 1389 reports of suspected ADRs were examined, accounting for a total of 4488 drug‐reaction pairs related to the paediatric use of PPIs. Fifty suspected ADRs were found to be statistically significant: 1 for dexlansoprazole, 9 for esomeprazole, 9 for lansoprazole, 5 for omeprazole, 14 for pantoprazole and 12 for rabeprazole. As shown in Table 3, statistically significant reactions related to omeprazole included choking (ROR 5.54; 95% CI, 1.04 to 29.56), pneumonia (ROR 3.61; 95% CI 1.41 to 9.20) and sputum discoloured (ROR 3.61; 95% CI 1.41 to 9.20). For esomeprazole, on the other hand, reactions with the highest ROR were gastrointestinal disorder (ROR 7.57; 95% CI 1.18 to 48.60) and constipation (ROR 4.74; 95% CI 2.25 to 9.98). A disproportion was found for chronic kidney disease associated with dexlansoprazole (ROR 106.21; 95% CI 14.82 to 761.40).

**TABLE 3:** Statistically significant drug‐reaction pairs for individual PPIs.

	Reactions	*n* ^a^	*n* ^b^	ROR	CI_low^c^	CI_up^d^	*p*
Dexlansoprazole	Chronic kidney disease	2	21	106.21	14.82	761.40	0.0004
Esomeprazole	Gastrointestinal disorder	5	2	7.57	1.18	48.60	0.0126
	Constipation	14	9	4.74	2.25	9.98	< 0.0001
	Confusional state	6	4	4.55	1.25	16.56	0.0192
	Insomnia	10	10	3.04	1.34	6.88	0.0132
	Agitation	13	14	2.82	1.42	5.61	0.0025
	Regurgitation	10	11	2.76	1.24	6.14	0.0079
	Decreased appetite	8	9	2.69	1.08	6.72	0.0390
	Gastrooesophageal reflux disease	12	15	2.43	1.21	4.89	0.0092
	Neutropenia	9	12	2.27	1.00	5.16	0.0281
Lansoprazole	Drug ineffective for unapproved indication	9	1	38.14	2.79	521.83	< 0.0001
	Therapy non‐responder	12	3	17.00	5.01	57.70	< 0.0001
	Deafness	4	2	8.42	1.18	59.97	0.0147
	Dehydration	6	5	5.06	1.53	16.72	0.0095
	Malaise	11	11	4.24	1.96	9.17	0.0011
	Drug interaction	5	6	3.51	1.05	11.72	0.0433
	Seizure	13	22	2.50	1.32	4.72	0.0035
	Urticaria	10	18	2.35	1.13	4.88	0.0133
	Rash	13	29	1.89	1.02	3.50	0.0267
Omeprazole	Choking	8	2	5.54	1.04	29.56	0.0172
	Pneumonia	13	5	3.61	1.41	9.20	0.0046
	Sputum discoloured	13	5	3.61	1.41	9.20	0.0046
	Dyspnoea	13	6	3.00	1.26	7.18	0.0097
	Diarrhoea	34	24	1.97	1.30	3.00	0.0050
Pantoprazole	Drug dependence	4	1	41.28	2.17	785.15	0.0003
	Heart disease congenital	4	2	20.64	2.89	147.30	0.0008
	Patent ductus arteriosus	3	2	15.44	1.86	128.24	0.0061
	Respiratory depression	3	2	15.44	1.86	128.24	0.0061
	Neonatal respiratory distress syndrome	4	3	13.75	2.67	70.82	0.0018
	Atrial septal defect	6	7	8.88	3.00	26.29	0.0005
	Foetal growth restriction	6	8	7.77	2.72	22.21	0.0008
	Leukopenia	3	4	7.72	1.52	39.26	0.0187
	No adverse event	3	6	5.14	1.19	22.22	0.0393
	Premature baby	16	41	4.11	2.39	7.08	< 0.0001
	Macule	7	18	4.03	1.71	9.47	0.0049
	Erythema	7	22	3.29	1.43	7.59	0.0118
	Rash papular	5	16	3.22	1.18	8.80	0.0337
	Dermatitis acneiform	7	24	3.02	1.32	6.90	0.0170
Rabeprazole	Drug withdrawal syndrome	4	1	80.99	4.24	1546.92	< 0.0001
	Drug withdrawal syndrome neonatal	17	5	73.29	30.47	176.29	< 0.0001
	Tremor	13	10	27.44	12.96	58.07	< 0.0001
	Sleep disorder	13	13	21.09	10.43	42.66	< 0.0001
	Crying	13	14	19.58	9.78	39.18	< 0.0001
	Premature baby	18	39	9.92	5.88	16.73	< 0.0001
	Dermatitis acneiform	8	23	7.14	3.25	15.71	< 0.0001
	Macule	6	19	6.43	2.58	16.02	0.0009
	Rash papular	5	16	6.33	2.31	17.37	0.0026
	Drug hypersensitivity	4	16	5.04	1.66	15.34	0.0135
	Rash macular	3	13	4.64	1.27	16.88	0.0383
	Erythema	4	25	3.22	1.11	9.37	0.0475

^a^ Number of drug‐reaction pairs for the PPI analysed.

^b^ Number of drug‐reaction pairs for all other PPIs.

^c^ Lower bound of the 95% confidence interval.

^d^ Upper bound of the 95% confidence interval.

## 4 Discussion

To our knowledge, the present study was the first to explore the safety of PPIs among children in a large spontaneous reporting database, such as EudraVigilance, over a 20‐year period. Between 2003 and 2022, a total of 1389 reports of suspected ADRs related to the administration of PPIs in patients aged 0–11 years were submitted to EudraVigilance and analysed in the current study. Reports mainly involved the use of omeprazole and esomeprazole, which were reported as suspect drugs in 47% and 27% of all reports, respectively. Based on the SPCs [28, 31], these are the only PPIs approved for use in patients aged 1 to 11 years, which is consistent with the greatest number of reports found associated with omeprazole and esomeprazole. Nevertheless, many reports retrieved in our study concerned infants younger than 1 year of age, for whom no PPIs are currently licensed in Europe. Dose‐related effects of PPIs in newborns have not yet been established [32], and the enzymes responsible for their metabolic clearance, cytochrome P450 (CYP) 2C19 and to a lesser degree CYP3A4, only reach mature activity at 5–6 months of life [33]. Given also the lack of demonstrated efficacy of PPIs in treating symptomatic GERD in otherwise healthy infants [10, 11], attention should be turned to the inappropriate and potentially harmful exposure to PPIs of this vulnerable population.

Based on our analysis, gastrointestinal disorders were among the most common adverse reactions occurring with PPI use in paediatric patients, with vomiting being the most frequently reported reaction for both esomeprazole (2.3%), lansoprazole (2.3%) and omeprazole (2.3%). In a 10‐year literature review of adverse effects reported in the use of GERD treatments in children, diarrhoea, nausea and constipation were routinely observed in patients treated with PPIs [34], which supports our finding of the gastrointestinal system being the most affected by adverse reactions following PPI administration. According to the SPCs, gastrointestinal disorders such as diarrhoea, nausea, vomiting, abdominal pain, flatulence and constipation are known as common ADRs (> 1/100, < 1/10) for all PPIs, except for pantoprazole for which they are rated as uncommon (> 1/1000, < 1/100). Gastric acid suppression caused by PPIs compromises the effectiveness of the gastric acid barrier, allowing increased bacterial colonisation of the intestinal tract [35]. Children with small bowel bacterial overgrowth show a higher mean symptom frequency score for bloating (1.33 ± 1.1 vs 0.29 ± 0.69, *p* = 0.001), abdominal pain (2.11 ± 0.93 vs 1.13 ± 0.81, *p* = 0.004), eructation (1.56 ± 0.88 vs 0.35 ± 0.75, *p* < 0.001) and flatulence (1.33 ± 1.23 vs 0.45 ± 0.81, *p* = 0.024) [36], which supports gastrointestinal side effects of paediatric PPI use. However, since small intestinal bacterial overgrowth induces symptoms similar to those for which PPIs are often prescribed [32], it is not always clear whether a gastrointestinal event is due to the drug treatment or to the disease itself [34]. Furthermore, considering that ADR ‘drug ineffective’ emerged as one of the most reported outcomes in our study, the persistence of gastrointestinal disorders might also suggest a limited effectiveness of PPIs, which is consistent with their questionable benefits in reducing paediatric GERD symptoms, especially in infants under 1 year of age [10, 11]. This aspect makes it even more important to appropriately prescribe these drugs (in any patient and even more in paediatrics) because an inappropriate prescription, in addition to not providing any clinical benefit, would expose the little patient to adverse reactions, which, as we have seen from our results, can also be life‐threatening.

Nearly 76% of all reports of suspected ADRs concerned one or more serious outcomes, including a total of 48 (3.5%) deaths. Of these, 17 were related to PPIs as the only suspect drug. In a review of 44 randomised controlled trials, adverse effects related to GERD treatments in children were usually mild in the PPI group [34], which is deeply in contrast to our findings. About 71% of the serious reports in our study involved multiple suspect drugs and/or concomitant drugs. So, as suggested by Dipasquale et al. [37], combination therapy might be positively linked to the seriousness of ADRs. Combined therapy of PPIs with other drugs may affect the risk of adverse outcomes as PPIs have the potential to provoke drug–drug interactions [38]. However, patients requiring the use of multiple medications are likely to have complex or multiple health conditions, for which clinical symptoms may be misinterpreted as ADRs. Furthermore, as EudraVigilance is a spontaneous reporting database, non‐serious adverse reactions are more likely to be under‐reported, leading to an overestimated proportion of serious outcomes. Given the significantly high percentage of serious reports found together with the inconsistency with available evidence, associations with ADR seriousness need to be further explored to prevent potential serious adverse effects of PPIs in the paediatric population.

According to the statistical analysis in our surveillance study, a disproportionality emerged for esomeprazole compared with other PPIs for many gastrointestinal outcomes, including gastrointestinal disorder, constipation, regurgitation, decreased appetite and gastroesophageal reflux disease. Although gastrointestinal manifestations are reported among the most frequent adverse reactions related to the paediatric use of PPIs [34, 37], our findings might suggest a greater association of such events with esomeprazole. Since gastroesophageal reflux disease is the condition for which PPIs are commonly prescribed and many reported gastrointestinal outcomes may be GERD‐related, these terms might reflect the therapeutic indication or, on the other hand, might suggest limited effectiveness of esomeprazole in treating GERD. Among other statistically significant reactions, pneumonia was found to be more frequently related to omeprazole. PPI use in young children was associated with increased risks of serious infections overall as well as the risk of infections in the lower respiratory tract (adjusted Hazard Ratio 1.22; 95% CI, 1.19 to 1.25) [39]. Furthermore, in a prospective study on the association between acid suppressive medications and infections, an increased risk of pneumonia was reported in GERD‐affected children treated with either ranitidine or omeprazole (Odds Ratio 6.39; 95% CI, 1.38 to 29.70) [40]. Nevertheless, evidence on the risk of pneumonia in children exposed to PPIs is still poor and conflicting [41, 42, 43], and further studies are needed to investigate a potential greater association with omeprazole versus other PPIs to protect paediatric patients at higher risk of respiratory infections.

Although PPIs are generally well tolerated, concerns have recently emerged about the use of PPIs and the potential development of allergic disorders in children [15]. In a large retrospective cohort study, exposure to PPIs during infancy was found to be associated with an increased risk for all major categories of childhood allergic diseases, including food allergy (adjusted hazard ratio 2.59; 95% CI, 2.25 to 3.00), medication allergy (1.84; 1.56 to 2.17), anaphylaxis (1.45; 1.22 to 1.73), allergic rhinitis (1.44; 1.36 to 1.52), asthma (1.41; 1.31 to 1.52), allergic conjunctivitis (1.15; 1.04 to 1.27) and urticaria (1.27; 1.17 to 1.38) [16]. In our surveillance study, the most commonly detected allergic conditions were urticaria, hypersensitivity and drug hypersensitivity. In addition to causing intestinal dysbiosis, antiulcer medications may enhance the development of allergic diseases by decreasing the breakdown of orally ingested proteins in the stomach. This facilitates immunoglobulin (Ig) E production in response to food and non‐food antigens and potentially leads to food and drug allergies in humans [44]. Among PPI‐related allergic conditions, evidence of a potential harmful association with asthma has recently been observed in children [16, 17, 18]. In a nationwide cohort study in Sweden, children and adolescents 17 years or younger who initiated PPI use had a 57% significantly increased risk of developing asthma compared with non‐initiators, with infants and toddlers having the highest risk [17]. Based on our analysis in EudraVigilance, a total of six patients reported asthma as a suspected adverse reaction to PPIs, mostly male and aged between 3 and 11 years. However, given the lack of data on paediatric patients exposed to PPIs, we were not able to estimate the incidence rate of asthma associated with PPI use in children. With regard to other allergic conditions, severe cutaneous adverse drug reactions (SCARs) have been reported in paediatric patients receiving PPIs, including six cases of toxic epidermal necrolysis, five of Stevens‐Johnson syndrome and four of drug reaction with eosinophilia and systemic symptoms. In two cases, the PPI was the only suspected drug, whereas in nine out of 15 cases, the suspected PPI reported was omeprazole. Healthcare professionals should be informed about the possible occurrence of this serious ADR in their young patients. SCARs are rare and serious hypersensitivity reactions, yet are associated with considerable morbidity and mortality, for which drug administration is the most common cause [45]. In a pharmacoepidemiologic study exploring the risk of drug hypersensitivity reactions in hospitalised patients treated with PPIs, severe drug‐induced skin reactions, including drug rash with eosinophilia and systemic symptoms and Stevens–Johnson syndrome/toxic epidermal necrolysis, were detected in association with PPI use [46]. However, the study population was limited to adults, and, to our knowledge, no paediatric studies have shown the occurrence of SCARs in association with PPI use. Since SCARs are potentially life‐threatening and a significant limitation to the safe use of drugs, further studies are needed to assess the risk of SCARs among paediatric patients receiving PPIs in the real clinical world.

The present surveillance study should be considered in light of some limitations. As EudraVigilance is designed as a spontaneous reporting system, reports of suspected ADRs may contained incomplete, inaccurate and/or coincident information, providing low‐quality data and also affecting duplicate detection. The inability to identify and merge duplicates, together with the potential for reporting the same adverse reaction under different terms, may provide an overestimated proportion of suspected ADRs and thus distort the actual frequency of ADRs in clinical practice. The main limitation of spontaneous reporting systems remains underreporting, which is expected to be substantial especially in the paediatric population. Collection of pharmacovigilance data should take into account that off‐label use of drugs is a common practice in paediatric patients [47] and that reasons for this under‐reporting may include fear of potential legal consequences in case of ADRs resulting from off‐label uses [48]. In addition, adverse reactions that are dependent on patient communication ability in newborns and infants might be less likely to be suspected and reported, which contributes to under‐reporting in younger children. In the absence of data on the exposure of paediatric patients to PPIs, we were not able to estimate incidence rates of reported suspected reactions but could only provide a frequency estimate on the total number of reactions regarding PPI use in children retrieved from EV. Finally, it is important to remember that low number of reports for some drugs or ADRs may limit the statistical power of the disproportionality analysis leading to an underestimation or misinterpretation of certain ADRs. Odds ratio does not allow to assess the causal relation between a suspect drug and a given adverse reaction, therefore requiring further pharmacoepidemiologic studies are more prone to support this type of comparison and to determine the true risk associated with the use of PPIs in the paediatric population. Despite its inherent limitations, surveillance studies based on spontaneous reports, such as ours, still remain an instrumental pharmacovigilance tool in detecting early safety warnings, having the potential to provide preliminary information on any ADRs from the real clinical setting. Given the scarcity of paediatric clinical trials along with their known limitations in generating safety data, spontaneous reporting may even be the only source of information on adverse reactions occurring in children, which supports the importance of conducting tailored pharmacovigilance research in the paediatric population [47]. Although PPIs are generally considered well‐tolerated and safe, safety concerns remain especially regarding potential long‐term side effects and prolonged use of these drugs in paediatric patients [49]. As PPIs have become the only antisecretory drugs for use in children since the EU‐wide suspension of all ranitidine products in 2020 [50], continuous monitoring of potential safety issues is warranted to ensure the safe use of PPIs in paediatric patients.

## 5 Conclusions

Our post‐marketing surveillance study has provided further evidence on the safety profile of PPIs in children. The most common adverse reactions reported in clinical practice with paediatric PPI use were gastrointestinal disorders and allergic reactions, consistent with the safety profile in the product license. However, there was a substantially high proportion of serious adverse reactions, as well as a few cases of rare and serious cutaneous ADRs. Considering the increasing use of PPIs among children, continuous monitoring will be critical for a comprehensive evaluation of the benefit/risk profile of PPIs in the paediatric population.

## Author Contributions

Substantial contributions to conception or design of the work: Domenico Motola, Greta Santi Laurini, Victoria Nikitina, Nicola Montanaro; the acquisition: Greta Santi Laurini; analysis: Domenico Motola, Victoria Nikitina, Nicola Montanaro; interpretation of data for the work: Greta Santi Laurini, Victoria Nikitina, Domenico Motola, Nicola Montanaro; drafting of the work: Greta Santi Laurini; revising the work critically for important intellectual content: Nicola Montanaro, Victoria Nikitina, Domenico Motola. All authors approved the submitted final version to be published. All authors agree to be accountable for all aspects of the work in ensuring that questions related to the accuracy or integrity of any part of the work are appropriately investigated and resolved.

## Ethics Statement

The authors have nothing to report.

## Consent

The authors have nothing to report.

## Conflicts of Interest

The authors declare no conflicts of interest.

## Supporting information


**Table S1** Age and sex of patients expressing suspected ADRs for individual PPIs.
**Table S2.** Suspected ADRs reported in EudraVigilance with frequency ≥ 5 for individual PPIs.
**Table S3.** Report of suspected ADRs related to deaths with PPIs as the only suspect drug.

## Data Availability

The data that support the findings of this study are available from the corresponding author upon reasonable request.
