# Supplementary material for: Safety of Proton Pump Inhibitor in Paediatrics: A Study Based on EudraVigilance Data
Source: Fundam Clin Pharmacol. 2025 Jul 7;39(4):e70036. doi: 10.1111/fcp.70036 (PMC12234264; doi:10.1111/fcp.70036)
Supplement: Supplementary file 1 — Table S1 Age and sex of patients expressing suspected ADRs for individual PPIs. Table S2. Suspected ADRs reported in EudraVigilance with frequency ≥ 5 for individual PPIs. Table S3. Report of suspected ADRs related to deaths with PPIs as the only suspect drug. [file FCP-39-0-s001.docx]

**Safety of proton pump inhibitor use in paediatrics: a pharmacovigilance study based on EudraVigilance data**

**Authors:** Greta Santi Laurini^1^, PharmD, Victoria Nikitina^1^, PharmD, Nicola Montanaro^2^, PhD, Domenico Motola^1^, PhD

1 Unit of Pharmacology, Department of Medical and Surgical Sciences, Alma Mater Studiorum University of Bologna, Bologna, Italy;

2 Former Professor of Pharmacology at the Alma Mater Studiorum University di Bologna.

**ORCID:**

Greta Santi Laurini: [https://orcid.org/0000-0002-7897-2956](https://orcid.org/0000-0001-6253-4014)

Victoria Nikitina: <https://orcid.org/0000-0002-0089-3043>

Nicola Montanaro: <https://orcid.org/0000-0002-5710-8077>

Domenico Motola: <https://orcid.org/0000-0001-6253-4014>

**Corresponding Author:** Prof Domenico Motola, Unit of Pharmacology, Department of Medical and Surgical Sciences, University of Bologna, via Irnerio 48, 40126, Bologna, Italy. E-mail: domenico.motola@unibo.it - Phone: +39-051-2091779

**Table S1** Age and sex of patients expressing suspected ADRs for individual PPIs

|  | Age | Sex | Subtotal | % | Total | % |
| --- | --- | --- | --- | --- | --- | --- |
| **Dexlansoprazole** | 0-1 month | Female | 1 | 33.33 | 1 | 33.33 |
|  |  | Male | 0 | 0.00 |  |  |
|  |  | Not specified | 0 | 0.00 |  |  |
|  | 2 months - 2 years | Female | 1 | 33.33 | 1 | 33.33 |
|  |  | Male | 0 | 0.00 |  |  |
|  |  | Not specified | 0 | 0.00 |  |  |
|  | 3-11 years | Female | 1 | 33.33 | 1 | 33.33 |
|  |  | Male | 0 | 0.00 |  |  |
|  |  | Not specified | 0 | 0.00 |  |  |
|  | Total |  | 3 | 100 | 3 | 100 |
| **Esomeprazole** | 0-1 month | Female | 45 | 11.87 | 80 | 21.11 |
|  |  | Male | 28 | 7.39 |  |  |
|  |  | Not specified | 7 | 1.85 |  |  |
|  | 2 months - 2 years | Female | 77 | 20.32 | 156 | 41.16 |
|  |  | Male | 76 | 20.05 |  |  |
|  |  | Not specified | 3 | 0.79 |  |  |
|  | 3-11 years | Female | 66 | 17.41 | 143 | 37.73 |
|  |  | Male | 76 | 20.05 |  |  |
|  |  | Not specified | 1 | 0.26 |  |  |
|  | Total |  | 379 | 100 | 379 | 100 |
| **Lansoprazole** | 0-1 month | Female | 8 | 3.24 | 23 | 9.31 |
|  |  | Male | 13 | 5.26 |  |  |
|  |  | Not specified | 2 | 0.81 |  |  |
|  | 2 months - 2 years | Female | 50 | 20.24 | 107 | 43.32 |
|  |  | Male | 52 | 21.05 |  |  |
|  |  | Not specified | 5 | 2.02 |  |  |
|  | 3-11 years | Female | 42 | 17.00 | 117 | 47.37 |
|  |  | Male | 72 | 29.15 |  |  |
|  |  | Not specified | 3 | 1.21 |  |  |
|  | Total |  | 247 | 100 | 247 | 100 |
| **Omeprazole** | 0-1 month | Female | 45 | 6.91 | 91 | 13,98 |
|  |  | Male | 41 | 6.30 |  |  |
|  |  | Not specified | 5 | 0.77 |  |  |
|  | 2 months - 2 years | Female | 119 | 18.28 | 286 | 43.93 |
|  |  | Male | 156 | 23.96 |  |  |
|  |  | Not specified | 11 | 1.69 |  |  |
|  | 3-11 years | Female | 117 | 17.97 | 274 | 42.09 |
|  |  | Male | 155 | 23.81 |  |  |
|  |  | Not specified | 2 | 0.31 |  |  |
|  | Total |  | 651 | 100 | 651 | 100 |
| **Pantoprazole** | 0-1 month | Female | 16 | 10.19 | 44 | 28.03 |
|  |  | Male | 16 | 10.19 |  |  |
|  |  | Not specified | 12 | 7.64 |  |  |
|  | 2 months - 2 years | Female | 21 | 13.38 | 42 | 26.75 |
|  |  | Male | 19 | 12.10 |  |  |
|  |  | Not specified | 2 | 1.27 |  |  |
|  | 3-11 years | Female | 24 | 15.29 | 71 | 45.22 |
|  |  | Male | 44 | 28.03 |  |  |
|  |  | Not specified | 3 | 1.91 |  |  |
|  | Total |  | 157 | 100 | 157 | 100 |
| **Rabeprazole** | 0-1 month | Female | 1 | 2.44 | 21 | 51.22 |
|  |  | Male | 4 | 9.76 |  |  |
|  |  | Not specified | 16 | 39.02 |  |  |
|  | 2 months - 2 years | Female | 4 | 9.76 | 5 | 12.20 |
|  |  | Male | 1 | 2.44 |  |  |
|  |  | Not specified | 0 | 0.00 |  |  |
|  | 3-11 years | Female | 1 | 2.44 | 15 | 36.59 |
|  |  | Male | 14 | 34.15 |  |  |
|  |  | Not specified | 0 | 0.00 |  |  |
|  | Total |  | 41 | 100 | 41 | 100 |

**Table S2** Suspected ADRs reported in EudraVigilance with frequency ≥ 5 for individual PPIs

|  | *Reactions* | *n* | | *%^a^* | |
| --- | --- | --- | --- | --- | --- |
| **Esomeprazole** | Vomiting | 26 | | 2.33 | |
|  | Drug ineffective | 16 | | 1.43 | |
|  | Constipation | 14 | | 1.25 | |
|  | Diarrhoea | 14 | | 1.25 | |
|  | Agitation | 13 | | 1.16 | |
|  | Gastrooesophageal reflux disease | 12 | | 1.07 | |
|  | Abdominal pain | 11 | | 0.98 | |
|  | Insomnia | 10 | | 0.90 | |
|  | Pyrexia | 10 | | 0.90 | |
|  | Regurgitation | 10 | | 0.90 | |
|  | Nausea | 9 | | 0.81 | |
|  | Neutropenia | 9 | | 0.81 | |
|  | Decreased appetite | 8 | | 0.72 | |
|  | Headache | 8 | | 0.72 | |
|  | Seizure | 8 | | 0.72 | |
|  | Premature baby | 7 | | 0.63 | |
|  | Pruritus | 7 | | 0.63 | |
|  | Rash | 7 | | 0.63 | |
|  | Urticaria | 7 | | 0.63 | |
|  | Abdominal pain upper | 6 | | 0.54 | |
|  | Acute kidney injury | 6 | | 0.54 | |
|  | Confusional state | 6 | | 0.54 | |
|  | Flatulence | 6 | | 0.54 | |
|  | Foetal growth restriction | 6 | | 0.54 | |
|  | Sleep disorder | 6 | | 0.54 | |
|  | Condition aggravated | 5 | | 0.45 | |
|  | Drug intolerance | 5 | | 0.45 | |
|  | Gastrointestinal disorder | 5 | | 0.45 | |
|  | Haematochezia | 5 | | 0.45 | |
|  | Hepatic cytolysis | 5 | | 0.45 | |
|  | Hepatic enzyme increased | 5 | | 0.45 | |
|  | Pancreatitis | 5 | | 0.45 | |
|  | Somnolence | 5 | | 0.45 | |
|  | Sputum discoloured | 5 | | 0.45 | |
|  | Thrombocytopenia | 5 | | 0.45 | |
|  | Weight decreased | 5 | | 0.45 | |
| **Lansoprazole** | Vomiting | 20 | | 2.31 | |
|  | Rash | 13 | | 1.50 | |
|  | Seizure | 13 | | 1.50 | |
|  | Therapy non-responder | 12 | | 1.39 | |
|  | Drug ineffective | 11 | | 1.27 | |
|  | Malaise | 11 | | 1.27 | |
|  | Lower respiratory tract infection | 10 | | 1.16 | |
|  | Pyrexia | 10 | | 1.16 | |
|  | Urticaria | 10 | | 1.16 | |
|  | Antimicrobial susceptibility test resistant | 9 | | 1.04 | |
|  | Drug ineffective for unapproved indication | 9 | | 1.04 | |
|  | Dermatitis acneiform | 8 | | 0.93 | |
|  | Minimum inhibitory concentration | 8 | | 0.93 | |
|  | Therapeutic response delayed | 8 | | 0.93 | |
|  | Chronic kidney disease | 7 | | 0.81 | |
|  | Condition aggravated | 7 | | 0.81 | |
|  | Erythema | 7 | | 0.81 | |
|  | Dehydration | 6 | | 0.69 | |
|  | Diarrhoea | 6 | | 0.69 | |
|  | Headache | 6 | | 0.69 | |
|  | Macule | 6 | | 0.69 | |
|  | Agitation | 5 | | 0.58 | |
|  | Drug interaction | 5 | | 0.58 | |
|  | Fatigue | 5 | | 0.58 | |
|  | Nausea | 5 | | 0.58 | |
|  | Rash macular | 5 | | 0.58 | |
|  | Rash papular | 5 | | 0.58 | |
|  | Somnolence | 5 | | 0.58 | |
| **Omeprazole** | Vomiting | 44 | | 2.33 | |
|  | Hypertrichosis | 35 | | 1.86 | |
|  | Diarrhoea | 34 | | 1.80 | |
|  | Drug ineffective | 22 | | 1.17 | |
|  | Abdominal pain | 18 | | 0.95 | |
|  | Pyrexia | 18 | | 0.95 | |
|  | Rash | 17 | | 0.90 | |
|  | Nausea | 15 | | 0.80 | |
|  | Dyspnoea | 13 | | 0.69 | |
|  | Headache | 13 | | 0.69 | |
|  | Pneumonia | 13 | | 0.69 | |
|  | Sputum discoloured | 13 | | 0.69 | |
|  | Abdominal pain upper | 12 | | 0.64 | |
|  | Gastrooesophageal reflux disease | 12 | | 0.64 | |
|  | Premature baby | 12 | | 0.64 | |
|  | Pancreatitis | 11 | | 0.58 | |
|  | Seizure | 11 | | 0.58 | |
|  | Infantile spitting up | 10 | | 0.53 | |
|  | Regurgitation | 10 | | 0.53 | |
|  | Somnolence | 10 | | 0.53 | |
|  | Urticaria | 10 | | 0.53 | |
|  | Erythema | 9 | | 0.48 | |
|  | Pancreatitis acute | 9 | | 0.48 | |
|  | Pruritus | 9 | | 0.48 | |
|  | Tachycardia | 9 | | 0.48 | |
|  | Choking | 8 | | 0.42 | |
|  | Death | 8 | | 0.42 | |
|  | Dermatitis acneiform | 8 | | 0.42 | |
|  | Flatulence | 8 | | 0.42 | |
|  | Hepatic function abnormal | 8 | | 0.42 | |
|  | Hypoglycaemia | 8 | | 0.42 | |
|  | Neutropenia | 8 | | 0.42 | |
|  | Oral administration complication | 8 | | 0.42 | |
|  | Pulmonary malformation | 8 | | 0.42 | |
|  | Respiratory distress | 8 | | 0.42 | |
|  | Abdominal discomfort | 7 | | 0.37 | |
|  | Abdominal distension | 7 | | 0.37 | |
|  | Acute kidney injury | 7 | | 0.37 | |
|  | Anaemia | 7 | | 0.37 | |
|  | Chronic kidney disease | 7 | | 0.37 | |
|  | Constipation | 7 | | 0.37 | |
|  | Crying | 7 | | 0.37 | |
|  | Drug hypersensitivity | 7 | | 0.37 | |
|  | Dysphagia | 7 | | 0.37 | |
|  | Gynaecomastia | 7 | | 0.37 | |
|  | Hyperhidrosis | 7 | | 0.37 | |
|  | Lung cyst | 7 | | 0.37 | |
|  | Oxygen saturation decreased | 7 | | 0.37 | |
|  | Toxicity to various agents | 7 | | 0.37 | |
|  | Adenovirus infection | 6 | | 0.32 | |
|  | Agitation | 6 | | 0.32 | |
|  | Condition aggravated | 6 | | 0.32 | |
|  | Cough | 6 | | 0.32 | |
|  | Decreased appetite | 6 | | 0.32 | |
|  | Fatigue | 6 | | 0.32 | |
|  | Gastric mucosal hypertrophy | 6 | | 0.32 | |
|  | Haematochezia | 6 | | 0.32 | |
|  | Hyperbilirubinaemia | 6 | | 0.32 | |
|  | Hypersensitivity | 6 | | 0.32 | |
|  | Insomnia | 6 | | 0.32 | |
|  | Macule | 6 | | 0.32 | |
|  | Malaise | 6 | | 0.32 | |
|  | Poor sucking reflex | 6 | | 0.32 | |
|  | Respiratory tract malformation | 6 | | 0.32 | |
|  | Transaminases increased | 6 | | 0.32 | |
|  | Abnormal behaviour | 5 | | 0.27 | |
|  | Agranulocytosis | 5 | | 0.27 | |
|  | Alanine aminotransferase increased | 5 | | 0.27 | |
|  | Aspartate aminotransferase increased | 5 | | 0.27 | |
|  | Bacterial infection | 5 | | 0.27 | |
|  | Cardiac murmur | 5 | | 0.27 | |
|  | Electrocardiogram QT prolonged | 5 | | 0.27 | |
|  | Epilepsy | 5 | | 0.27 | |
|  | Haematemesis | 5 | | 0.27 | |
|  | Hallucination | 5 | | 0.27 | |
|  | Hepatitis | 5 | | 0.27 | |
|  | Hyponatraemia | 5 | | 0.27 | |
|  | Hypotension | 5 | | 0.27 | |
|  | Juvenile idiopathic arthritis | 5 | | 0.27 | |
|  | Mydriasis | 5 | | 0.27 | |
|  | Pancreatitis haemorrhagic | 5 | | 0.27 | |
|  | Rash papular | 5 | | 0.27 | |
|  | Sleep disorder | 5 | | 0.27 | |
|  | Torus fracture | 5 | | 0.27 | |
|  | Toxic epidermal necrolysis | 5 | | 0.27 | |
| **Pantoprazole** | Premature baby | 16 | | 4.00 | |
|  | Dermatitis acneiform | 7 | | 1.75 | |
|  | Erythema | 7 | | 1.75 | |
|  | Macule | 7 | | 1.75 | |
|  | Atrial septal defect | 6 | | 1.50 | |
|  | Foetal growth restriction | 6 | | 1.50 | |
|  | Vomiting | 6 | | 1.50 | |
|  | Drug ineffective | 5 | | 1.25 | |
|  | Rash papular | 5 | | 1.25 | |
| **Rabeprazole** | Premature baby | 18 | | 8.37 | |
|  | Trismus | 18 | | 8.37 | |
|  | Drug withdrawal syndrome neonatal | 17 | | 7.91 | |
|  | Crying | 13 | | 6.05 | |
|  | Sleep disorder | 13 | | 6.05 | |
|  | Tremor | 13 | | 6.05 | |
|  | Muscle tone disorder | 12 | | 5.58 | |
|  | Dermatitis acneiform | 8 | | 3.72 | |
|  | Macule | 6 | | 2.79 | |
|  | Rash papular | 5 | | 2.33 | |
| ^a^ Of the total number of suspected ADRs for individual PPIs. | |  |  | |  |

**Table S3** Report of suspected ADRs related to deaths with PPIs as the only suspect drug

|  | *Report* | *Patient sex* | *Patient age* | *Reaction list* | *Concomitant drugs* | *Indication* |
| --- | --- | --- | --- | --- | --- | --- |
| **Esomeprazole** | 1 | Male | 2 months - 2 years | Product use in unapproved indication  Sudden infant death syndrome | Not reported | Gastrooesophageal reflux disease |
|  | 2 | Female | 3-11 years | Hepatic fibrosis Hepatotoxicity Hyperammonaemia Off label use | Not reported | Not available |
|  | 3 | Not specified | 0-1 month | Death Product administered to patient of inappropriate age Vomiting | Not reported | Not available |
|  | 4 | Female | 3-11 years | Asthenia Death Feeding disorder Haematochezia Pneumonia aspiration Product administered to patient of inappropriate age | Not reported | Not available |
|  | 5 | Female | 3-11 years | Cardiac arrest Laryngeal oedema Off label use Respiratory depression Seizure Vomiting | Amikacin Ceftriaxone | Gastrointestinal disorder prophylaxis |
| **Lansoprazole** | 6 | Female | 2 months - 2 years | Sudden infant death syndrome | Not reported | Gastrooesophageal reflux disease |
|  | 7 | Male | 2 months - 2 years | Ascites Cardiopulmonary failure Clostridium difficile colitis Condition aggravated Diarrhoea Haemodynamic instability Product use issue Pyrexia Retroperitoneal haemorrhage Vomiting | Not reported | Gastrooesophageal reflux disease |
|  | 8 | Male | 0-1 month | Congenital genital malformation male Foetal death | Amoxicillin Clarithromycin | Helicobacter gastritis |
|  | 9 | Female | 3-11 years | Abdominal pain upper Acute hepatic failure Haemophagocytic lymphohistiocytosis Jaundice Lip discolouration Lip swelling | Bacillus Calmette-Guérin vaccine | Not available |
| **Omeprazole** | 10 | Male | 3-11 years | Abdominal distension Disseminated intravascular coagulation Gastrointestinal haemorrhage Pyrexia Respiratory distress Shock Status epilepticus | Not reported | Not available |
|  | 11 | Female | 2 months - 2 years | Acidosis Anuria Cardio-respiratory arrest Choking Dyspnoea Hypotonia Hypoxia Incorrect product formulation administered Pupil fixed Unresponsive to stimuli | Ceratonia | Gastrooesophageal reflux disease |
|  | 12 | Male | 2 months - 2 years | Hypoplastic left heart syndrome | Not reported | Not available |
|  | 13 | Male | 0-1 month | Death neonatal Foetal exposure during pregnancy | Not reported | Dyspepsia |
|  | 14 | Female | 2 months - 2 years | Aorto-oesophageal fistula Cardio-respiratory arrest Haematemesis | Octreotide | Not available |
|  | 15 | Female | 2 months - 2 years | Death Drug ineffective | Amikacin | Sepsis |
|  | 16 | Female | 3-11 years | Death Drug ineffective | Gentamicin Vancomycin | Sepsis |
| **Pantoprazole** | 17 | Not specified | 0-1 month | Heart disease congenital | Amoxicillin and clavulanic acid Clarithromycin Magaldrate Dextromethorphan, pseudoephedrine and paracetamol Aluminium hydroxide and magnesium hydroxide | Duodenitis Gastritis |
